# Mitochondrial Stress Signaling Promotes Cellular Adaptations

**DOI:** 10.1155/2014/156020

**Published:** 2014-01-22

**Authors:** Jayne Alexandra Barbour, Nigel Turner

**Affiliations:** Department of Pharmacology, School of Medical Sciences, University of New South Wales, Sydney, NSW 2052, Australia

## Abstract

Mitochondrial dysfunction has been implicated in the aetiology of many complex diseases, as well as the ageing process. Much of the research on mitochondrial dysfunction has focused on how mitochondrial damage may potentiate pathological phenotypes. The purpose of this review is to draw attention to the less well-studied mechanisms by which the cell adapts to mitochondrial perturbations. This involves communication of stress to the cell and successful induction of quality control responses, which include mitophagy, unfolded protein response, upregulation of antioxidant and DNA repair enzymes, morphological changes, and if all else fails apoptosis. The mitochondrion is an inherently stressful environment and we speculate that dysregulation of stress signaling or an inability to switch on these adaptations during times of mitochondrial stress may underpin mitochondrial dysfunction and hence amount to pathological states over time.

## 1. Introduction

Approximately 1.45 billion years ago, gram negative bacteria were engulfed by primitive eukaryotic cells giving rise to the mitochondrion [1–3]. However, the complex relationship between this organelle and its host is not fully understood, and the critical role that mitochondria play in various disease states has only been appreciated in recent years. Nuclear encoded proteins coordinate with mitochondrially encoded proteins for the biogenesis and maintenance of the complete mitoproteome. In return, mitochondria produce 90% of the cells ATP. Despite this elegant symbiosis, the inherent differences between mitochondria and the rest of the cell can lead to complications that may ultimately have pathological consequences. For instance, mtDNA release can stimulate an inflammatory response in the host cell [4]. Mitochondria possess a harsh protein folding environment, due to the high levels of reactive oxygen species (ROS), and the fact that more than 99% of mitochondrial proteins need to be transported from the cytosol into the mitochondria and correctly folded. In addition to proteotoxic stress, mitochondria are highly susceptible to DNA mutations from ROS and a high DNA replication error rate, which is confounded by less sophisticated DNA repair mechanisms [5].

**Figure 1:**
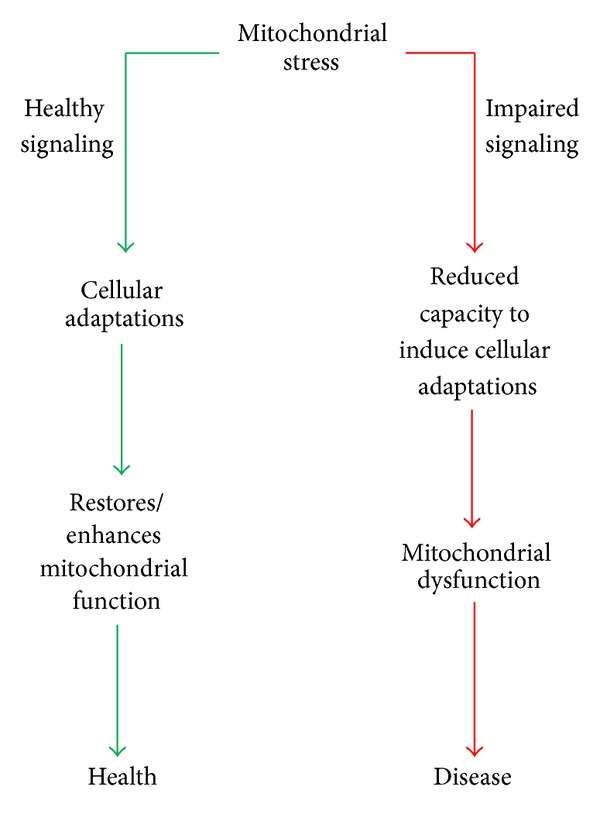
Hypothesis of responses to mitochondrial stress in health and disease. In times of mitochondrial stress, signals are sent to the cell which promote cellular adaptations that restore or possibly enhance mitochondrial function to maintain health (green arrows). In situations where mitochondrial stress signals are not relayed to other parts of the cell or the cell does not respond stress signals (red arrows), there is a failure for cellular adaptations which may consequently result in mitochondrial dysfunction and the development of disease.

Being the site of programmed cell death and energy metabolism, the cells survival is ultimately dependent on precise coordination between mitochondria and the rest of the cell. Consequently there are a number of mitochondrial stress signals that are communicated to the rest of the cell that stimulate cellular adaptions, which support this organelle-host symbiotic relationship. This is an emerging area in mitochondrial biology that has not been well studied to date. We propose that inability for the cell to perceive and respond to mitochondrial stress may be a platform for mitochondrial dysfunction (Figure 1). Mitochondrial dysfunction is likely to be at least partly involved in the aetiology of complex diseases of ageing, including Parkinson's disease (PD) [6], Alzheimer's disease (AD) [7], pancreatic *β*-cell failure [8], and insulin resistance (IR) [9], as well as the ageing process itself [10]. As such, it is critical to better understand the molecular mechanisms coordinating mitochondrial stress signaling with cellular adaptations.

Much of the previous literature linking mitochondrial dysfunction with disease has focused on the linear view that mitochondrial stress causes damage, which subsequently causes disease. However, mitochondria respond to various stresses by inducing a complex array of cellular responses and adaptations to reduce the impact of subsequent stressors. The purpose of this review is to summarise the literature surrounding cellular adaptations and quality control processes to cope with mitochondrial stress, which may be potential pharmacological targets.

## 2. Mitochondrial Stress Signals

**Figure 2:**
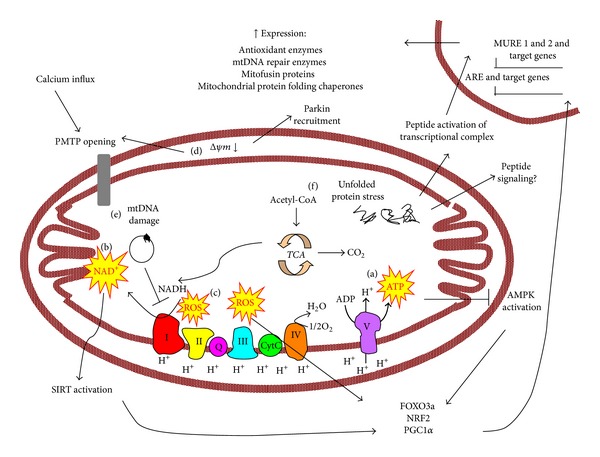
Schematic summary of mitochondrial stress signaling and cellular adaptations. ATP (a), NAD^+^ (b), and ROS (c) are outputs of the electron transport chain and oxidative phosphorylation that may function as stress signals. NAD^+^ can activate sirtuins (SIRT) and increased AMP/ATP ration can activate AMPK, which activate transcription of antioxidant defences, mitochondrial DNA repair enzymes, and other target genes important in mitochondrial biogenesis and metabolism through transcription factors FOXO3a, NRF2, and the transcriptional coactivator PGC1*α*. ROS can also directly activate these transcriptional regulators. Mitochondrial DNA (mtDNA) damage can potentiate oxphos dysfunction (e) and hence lead to the above responses. Loss of innermitochondrial membrane potential (d) can lead to PMTP opening or parkin recruitment and hence mitophagy. Through peptide export, unfolded protein stress (f) can activate a transcriptional complex which acts on the MURE1, MURE2, and CHOP elements to induce the transcription of mitochondrial protein folding chaperones.

It is clear that mitochondria are particularly vulnerable to endogenous stress and are major sensors of environmental stress, such as diet and toxic substances. Mitochondria signal stress by membrane depolarization, alterations in adenine nucleotide levels, ROS production, Ca^2+^ fluxes, permeability transition pore opening, and perhaps secretion of proteins/peptides (Figure 2). Here we will discuss how different stress signals promote the development of cellular adaptations, through retrograde signalling from the mitochondria to the nucleus, posttranslational modifications or activation of proteins and other mechanisms.

### 2.1. Impaired Oxidative Phosphorylation

Oxidative phosphorylation (oxphos) is the process whereby electrons gained during substrate oxidation are transported along the electron transport chain (ETC), coupled with the pumping of protons across the innermitochondrial membrane and the generation of a proton gradient, which is used as the driving force for ATP production at complex V (ATP synthase) in conjunction with oxygen consumption at complex IV. Defective oxphos reduces bioenergetic capacity and is a sign of cellular stress. Perturbations in oxphos can lead to reduced ATP production, changes in redox status, innermitochondrial membrane depolarization, and excessive reactive oxygen species (ROS) generation.

#### 2.1.1. Alterations in Energy Intermediates

*ATP Levels*. Oxphos is a multistep process that effectively results in ATP production from fuel substrates. ATP is a major output of oxphos function (Figure 2(a)) and hence the level of ATP and the ratio of ATP with other adenine nucleotides are a signal of mitochondrial function as well as cellular energy charge. During conditions of compromised mitochondrial function there are shifts in the ratio of adenine nucleotides and the best characterised cellular adaptation of an increased AMP/ATP ratio, resulting from defective oxphos, is the activation of the enzyme AMP-activated protein kinase (AMPK). This is a key energy-sensing kinase that reprograms cellular metabolism through phosphorylation of numerous target proteins.

Through alterations in the activity of downstream targets, AMPK activation rapidly stimulates a shift to catabolic metabolism and over the longer term increases mitochondrial biogenesis and oxidative capacity. Stimulation of AMPK has been shown to increase the expression of PGC1*α* and metabolic enzymes in skeletal muscle [11, 12] with increased fatty acid oxidation [12, 13] and glycogen synthesis [13]. Consistent with an important role for AMPK in energy transduction, oxidative capacity is reduced in myocardial tissue when AMPK activity is lost [14].

In addition to regulating mitochondrial substrate oxidation, AMPK has other effects on mitochondrial parameters. Metformin treatment activates AMPK activity in conjunction with inducing PGC1*α* and mitofusin protein 2 (Mfn2) protein expression in myocardial tissue [15]. A gain of function AMPK mutation in skeletal muscle has also been reported to increase the expression of mitochondrial fusion/fission proteins, Mfn2, optic atrophy 1 (OPA1), and dynamin-related protein 1 (Drp1) [16] which implicates AMPK in the regulation of mitochondrial dynamics as well as content. Furthermore, AMPK functionally prevents high-glucose induced mitochondrial fission in endothelial cells [17], highlighting that AMPK activity promotes mitochondrial quality control processes, as well as stimulating mitochondrial metabolism. In line with this, AMPK may function in the regulation of mitophagy, the autophagic clearance of mitochondria, through phosphorylation of an autophagy gene [18]. These latter two functions suggest AMPK influences quality control processes as well as regulation of mitochondrial oxidative metabolism.

*NAD* ^*+*^ *Levels*. In addition to ATP production, mitochondrial oxphos involves electrons being stripped out of fuel substrates and carried on NAD^+^, making NAD^+^ a signal of mitochondrial oxphos function (Figure 2(b)). The NAD^+^/NADH ratio, which signals redox status of the cell, can in turn be altered with changes in substrate flux and oxphos function. The redox ratio has been known for a long time to be an important feedback regulator of substrate metabolism, but in recent years it has become evident that NAD^+^ directly influences the activity of a number of enzymes that may affect quality control processes.

NAD^+^ activates Poly(ADP-ribose) polymerase 1 (PARP1) which is important for maintenance and repair of genomic DNA [19] and also stimulates transcription of NRF1 [20]. These dual actions of PARP1 activation suggest that low-grade bioenergetic stress and subsequent changes in the redox ratio may enhance the coordination of nuclear genomic integrity with regulation of mitochondrial function.

Another important group of enzymes regulated by changes in the NAD^+^/NADH ratio are the sirtuin (SIRT) family of NAD^+^-dependent deacetylases. Through alterations in posttranslational modifications (e.g., acetylation) on lysine residues, SIRT proteins influence a number of metabolic processes and cellular functions.

SIRT1 is known to regulate mitochondrial function in an anterograde, or nucleus to mitochondria fashion, signaling in response to redox status [21]. SIRT1 is activated by an increase in NAD^+^ and via deacetylation of the master regulator of mitochondrial biogenesis, PGC1*α*, and has been reported to increase mitochondrial bioenergetic capacity [22, 23]. In addition to regulating mitochondrial quantity, SIRT1 has been reported to regulate mitochondrial quality in response to redox status, by stimulating mitophagy [24]. SIRT3 is another important member of the sirtuin family likely to regulate mitochondrial function in response to redox status. SIRT3 is localised in the mitochondrion and has been shown to influence mitochondrial function more directly by deacetylating a range of enzymes involved in metabolic pathways in mitochondria [25, 26]. Alterations in SIRT3 activity can alter the response to stress pathways involving the MPTP and SIRT3 may also function in ameliorating oxidative stress in response to mitochondrial redox status, as it deacetylates and activates the antioxidant enzyme MnSOD [27], as well as FOXO3a [28], a transcription factor which promotes cellular antioxidant defences, which is described in more detail below.

#### 2.1.2. Mitochondrial Membrane Depolarization

In addition to changes in ATP and nutrient intermediates, alterations in oxphos can induce a stress response via changes in membrane potential. More specifically, the innermitochondrial membrane becomes polarized during oxphos and loss of the membrane potential or depolarization can act as a signal (Figure 2(d)), promoting mitophagy and mitochondrial permeability transition pore (MPTP) opening. These processes and their potential role in pathological states are discussed below.

*Mitophagy*. Mitochondrial depolarization can induce lysozomal degradation of mitochondria in a process called mitophagy. A major pathway mediating mitophagy has been identified which involves the PD associated proteins PTEN-induced putative kinase 1 (PINK1) and parkin. Parkin is an E3 ubiquitin ligase which translocates to mitochondria upon membrane depolarization in a PINK1-dependent process [29]. PINK1 actually binds to depolarized mitochondria, which recruits parkin to ubiquitinylated mitochondrial proteins [30]. Mitophagy is a quality control pathway which enables defective mitochondria to be cleared without impeding cell survival.

Defective mitophagy coincides with cell models of neurological diseases including AD [31] and PD [32], as well as pancreatic *β*-cell dysfunction [33], cardiomyopathy [34], and hepatic IR [35]. These findings strongly implicate reduced capacity to clear defective mitochondria with complex disease. However, the lack of mammalian *in vivo *models in this area means that it is unclear if these cellular effects are physiologically relevant or whether it is cause or consequence of disease. Another feature of these studies that makes it difficult to discern the relevance of reduced autophagic clearance of mitochondria in the aetiology of chronic disease is that defective mitophagy is usually defined as reduced parkin translocation in response to the uncoupling agents CCCP/FCCP under nonphysiological circumstances [36, 37]. Little research exists on levels of basal mitophagy in disease models. One study used colocalisation of mitochondria and lysozomes under normal state to signify basal mitophagy and found a reduction in basal mitophagy in fibroblasts derived from human patients with a mutation in PD associated protein DJ-1 [38], suggesting that impaired mitophagy may be a feature of human Parkinsonism. Further investigation of basal mitophagy *in vivo *in mammalian disease models would greatly facilitate insight into the role of defective mitophagy in disease development.

In all, mitophagy is a quality control process that enables adaptation to oxphos stress and may be protective against complex disease development. However, it is not currently understood how this process is switched on during physiologically normal mitochondrial membrane depolarization.

*Permeability Transition Changes*. Membrane depolarization can also initiate cell death by opening of the MPTP. This pore is a protein complex of VDAC, cyclophilin D (CypD), and adenine nucleotide translocase that can open and close and allows small substances to cross the mitochondrial membrane in response to membrane depolarization [39]. Opening of the MPTP causes mitochondrial swelling which can lead to cell death. Opening can be caused by ROS, mitochondrial calcium influxes from the ER, as well as membrane depolarization, but other factors may also influence the susceptibility of the MPTP to open in response to these stimuli [40]. Increased susceptibility of opening of the MPTP is linked with pathological states [41] and conversely, delayed response or inhibition of MPTP is associated with cell survival and reduced neurodegeneration [42].

Mitochondrial perturbations can lead to MPTP resistance as a cellular adaption. MPTP opening can be inhibited by NAD^+^, a signal of low energy status by SIRT3 deacetylation of CypD [43]. Inhibition of MPTP opening by CypD deficiency may further increase membrane depolarization by induction of uncoupling proteins, which have been proposed to offset high-fat diet induced obesity by increasing basal metabolic rate [44].

The phospholipid composition of the mitochondrial membrane also affects PMTP opening and now there is emerging evidence indicating that fatty acids and ceramides play a role in regulating the activity of MPTP which links lipids with mitochondrial dysfunction. Omega 3 fatty acid incorporation into the mitochondrial membrane delays MPTP opening [45]. Ceramides, a type of sphingolipids that are associated with IR, may disrupt healthy mitochondrial signaling by sensitizing the MPTP [46–48]. This suggests that a possible mechanism by which ceramides elicit IR is through potentiation of mitochondrial stress.

Membrane depolarization may result in either mitophagy or apoptosis depending on the extent of damage, which improves cellular or whole body functioning by cleansing damaged organelles or cells, respectively.

#### 2.1.3. Mitochondrial Reactive Oxygen Species Production

Mitochondria are a major site of ROS production (Figure 2(c)), as byproducts of normal oxphos function which can be further increased when there are oxphos defects [49]. ROS can readily diffuse out of the mitochondrial membrane and elicit cellular responses, making them active signaling molecules through activation of redox sensitive proteins, including transcription factors and transcriptional coactivators, making them key players in eliciting retrograde signaling.

Oxidative damage has been linked with a number of complex diseases, including neurological disorders [50, 51], cardiovascular diseases [52], and insulin resistance [53]. Furthermore, human polymorphisms in the antioxidant enzymes MnSOD and GPX are associated with increased risk of multiple cancers [54–57], cardiovascular disease [58], and diabetes [59].

Much of the literature surrounding ROS induced damage has incubated cultured cells in supraphysiological concentrations of exogenous hydrogen peroxide or has used high doses of pharmacological inhibitors of complex I and III for short periods of time [60–62]. These are major chemical insults that induce extensive cell damage and apoptosis and do not model endogenous, physiologically normal levels of mitochondrial ROS production during chronic mitochondrial stress. For instance, when oxphos dysfunction was modelled by DNA gamma polymerase mutation, resulting in complex I and III defects and an accompanying increase in ROS production, there was no decrease in cell survival because the cells were rescued by induction of selenoproteins GSH and GPX [63]. This was mediated by the zinc finger protein ZNF143 which is required for transcription of a protein required for the incorporation of selenocysteine into selenoproteins [63]. Another disease-related process which involves ROS is that of preconditioning, where a mild insult induces adaptations that protect against subsequent stresses. For example, low-grade ROS plays a role in inducing PKC epsilon translocation, which is involved in neuroprotective ischemic preconditioning, whereas larger ROS insults activate PKC delta, which causes apoptosis and neurodegeneration [64]. In line with these findings in neurons, mitochondrial ROS also have preconditioning roles in cardiomyocytes [65].

Extending on the idea that oxidative stress is involved in preconditioning phenomena, there is evidence that mild mitochondrial ROS stressors may actually improve lifespan, through a process termed “mitochondrial hormesis” (mitohormesis). This theory describes the link between low-grade mitochondrial stressors and enhanced cellular function [66]. Mitohormesis has been proposed to increase life span in *C. elegans* in response to low-grade arsenic exposure [67] and low-glucose availability [68]. Low-grade mitochondrial stressors have also been reported to protect neuronal cells against a secondary large stress by maintaining mitochondrial membrane potential [69], and low-dose complex I inhibition improves mitochondrial capacity and antioxidant defences in neuronal cells [70], implicating mitohormesis in neuroprotection. Although mitohormesis has been demonstrated to increase lifespan in *C. elegans* and improve functioning in mammalian cell culture, *in vivo* mammalian studies are lacking.

There are other examples where ROS induces cellular adaptations to clear subsequent ROS insults or improve mitochondrial functioning, which highlights an important negative feedback role for these molecules. One major mechanism that switches on antioxidant defences in the cell is the activation of antioxidant response element (ARE). ARE is a *cis*-acting enhancer sequence that controls expression of a variety of antioxidant enzymes. A ROS-mediated signaling cascade that results in activation of this enhancer has been identified, which provides evidence for cellular adaptation in response to ROS signals, where nuclear respiratory factor 2 (NRF2) transcription factor activates the ARE enhancer to switch on an antioxidant gene program [71]. Oxidation of lipids, especially membrane lipids, is one of the mechanisms proposed in the free radical theory of ageing, but products of lipid oxidation may actually function in cell signaling to cause adaptations. For instance, the oxidised lipid metabolite 4-hydroxynonenal promotes NRF2 activation of the ARE inducing expression of antioxidant genes [72]. In addition to ARE, ROS activates the transcription factor FOXO3a [73], which induces expression of MnSOD [74] catalase [75] and PrxIII [76] which have antioxidant functions in the mitochondria. Overall, these responses are protective against oxidative damage suggesting that low-grade ROS constitutes an important stress signal that can lead to adaptations that ameliorate a major insult later and protect against disease processes.

ROS not only induce antioxidant responses in a negative feedback type mechanism, but also can stimulate cellular adaptations that improve mitochondrial capacity in general. ROS increases mitochondrial biogenesis and mitochondrial DNA content in fibroblasts [77, 78]. Hydrogen peroxide treatment increases peroxisome proliferator-activated receptor gamma coactivator 1*α* (PGC1*α*) and PGC1*β* promoter activity and expression, and in addition to stimulating mitochondrial biogenesis, these transcriptional coactivators also stimulate the expression of multiple antioxidant defences [79, 80]. *In vivo* overexpression of pgc1*β* in rat skeletal muscle resulted in increased expression of antioxidant enzymes and an overall reduction in oxidative damage [81], highlighting its role in inducing antioxidant defenses. Nuclear respiratory factor 1 (NRF1) is a transcription factor associated with mitochondrial biogenesis and was found to be activated and subsequently activate mitochondrial transcription factor A (TFAM) in a redox-dependent pathway [82], which is again consistent with the notion that nontoxic levels of ROS can improve mitochondrial function. Additionally, the autophagy gene family Atg14 are regulated by ROS [83] and hence there may even be a role of mitochondrial ROS in stimulating mitophagy. Collectively these findings suggest that ROS are important signalling molecules that not only stimulate the expression of antioxidant systems to protect against future oxidative insults, but also can modify other aspects of mitochondrial function.

Overall, there has been a large focus in the literature on the effects of exogenous ROS *in vitro*. Increased understanding of the mechanisms that cells use to respond to mitochondrial oxidative stress *in vivo* is less thoroughly investigated to date and may be relevant in understanding the pathogenesis of complex disease, particularly as a reduced capacity to mount and antioxidant response is linked with a number of disease states [55, 56, 58, 59], as well as the ageing process [84].

### 2.2. Mitochondrial Morphology Transitions Are Important Responses to Mitochondrial Stress Signals

Mitochondria are dynamic organelles that constantly undergo morphological changes in the opposing processes of fusion and fission. In fusion, multiple mitochondria form one elongated mitochondrion, whereas, in fission, a mitochondrion generates small, fragmented mitochondria. Mitochondrial fission and division occurs when Drp-1 is recruited from the cytosol to divide the mitochondrion [85]. Fusion of mitochondria involves mitofusin proteins 1 and 2 (Mfn1 and Mfn2) anchoring outer mitochondrial membranes and OPA1 anchoring the innermitochondrial membranes [85].

Changes in mitochondrial morphology are known to occur in response to mitochondrial stressors and may be a cellular adaptation to regulate cell survival. The pro-survival protein Pim-1 and apoptotic protein PUMA have opposing roles in Drp-1 localisation to the mitochondria [86], suggesting that alterations in mitochondrial dynamics may be an integral part of the apoptotic program. In general, mitochondrial fusion occurs in response to mitochondrial stress and elongated mitochondria are thought to be pro-survival by increasing resistance to apoptosis and mitophagy. Mitochondrial elongation can be induced by ROS [87] as well as increase resistance to ROS [88], suggesting another feedback mechanism by which signals may allow the cell to adapt to mitochondrial oxidative stress. Mitochondrial fission, on the other hand, occurs in response to major stressors and primes mitochondria for mitophagy and apoptosis. Major stressors which promote mitochondrial fission and hence fragmentation include cyclosporine A [89] as well as excessive ceramides in yeast [90] and cardiomyocytes [91]. Additionally, short, high-dose incubations of hydrogen peroxide [92] and palmitate [93] induce mitochondrial fragmentation in C2C12 myotubes. Mitochondrial fission can increase mitochondrial uncoupling to ameliorate ROS production [94] as well as stimulate mitophagy [95, 96] and apoptosis [97–100] in order to let the cell adapt and respond to these major stressors.

Dysregulation of mitochondrial quality control processes has been implicated in pathological states and ageing. Down-regulation of Drp1 and mitochondrial elongation can be caused by a pharmacological DNA damage inducer [88] and cellular senescence [101], which mechanistically links ageing with reduced mitochondrial bioenergetics. Overexpression of AD associated protein, Tau, prevents Drp1 localisation to mitochondria [102] demonstrating that localisation as well as expression of mitochondrial morphology regulators is important in cellular quality control. Metabolic health may also be influenced by alterations in mitochondrial morphology with skeletal muscle expression of Mfn-2 being increased with exercise [103] and downregulated in obese mice [104] suggesting a link between energy metabolism and mitochondrial morphology transitions.

Overall the evidence in the literature indicates that the inherent plasticity in mitochondrial morphology provides the capacity for changes in this organelle in response to the prevailing environmental conditions, which provides a platform for rapidly changing bioenergetics and apoptotic state of the mitochondrion in response to specific stress signals.

### 2.3. Mitochondrial Genomic Stress Signals DNA Repair

Mitochondrial DNA (mtDNA) is double stranded and organised into a circular structure which encodes 37 genes, of which 13 are subunits of the ETC, 2 are rRNAs, and 22 are tRNAs [105]. mtDNA is susceptible to DNA damage and mutations from replication error, ROS, and basic DNA repair machinery [5, 106]. As mtDNA encodes for critical subunits of the ETC, mtDNA mutations often result in defective oxphos and increased ROS production [107] and hence act as a mitochondrial stress signal (Figure 2(e)).

mtDNA repair enzymes play an important role in responding to different stresses with increased activity of these enzymes that are protecting against oxidative stress induced apoptosis [108], palmitate induced IR and ROS production [109], and cardiac fibrosis [110]. Conversely, deficits in mitochondrial DNA repair have been shown to potentiate neurodegeneration [111] and age-related macular degeneration [112], which emphasises the potential importance of dysregulated cellular adaptation to mtDNA damage in the pathogenesis of disease.

Interestingly, many of the cellular adaptations to oxphos stress also lead to increased expression of mtDNA repair enzyme 8-oxoguanine glycosylase (OGG1), the major mitochondrial DNA repair enzyme. For example, the NRF2, part of the ROS induced antioxidant adaptation, can bind to the OGG1 promoter region to induce OGG1 expression [113], reinforcing the notion that, in response to mitochondrial stress, multiple cellular repair and adaptive responses are induced. The antioxidant enzyme MnSOD can also interact with DNA polymerase gamma to promote repair of mtDNA lesions [114], further linking antioxidant defences with mtDNA repair. There may also be a role for mitochondrial dynamics and degradation in mtDNA damage adaptation. Knockdown of mitochondrial fission, fusion, and mitophagy genes results in reduced capacity to clear mtDNA lesions [115] which reveals a role for mitophagy and morphology transitions in mtDNA cleansing. Mitochondrial OGG1 activity is also increased by exercise [116] which parallels upregulation of other mitochondrial quality control processes with physical activity.

Thus, while mtDNA does not have the same level of protection and repair as nDNA, there are a number of feedback mechanisms in place to respond to mitochondrial genotoxic stress.

### 2.4. Mitochondrial Proteotoxic Stress Signaling

It is well known that aberrant protein folding and subsequent formation of toxic oligomeric intermediates, such as amyloids, are implicated in chronic diseases of ageing. Given that mitochondrial dysfunction is also widely implicated in complex diseases of ageing and mitochondria are vulnerable to proteotoxic stress, it is possible that mitochondrial specific protein misfolding is a component of chronic disease. Consistent with this idea, accumulation of *β*-amyloid specifically in the mitochondrial fraction has been associated with severe mitochondrial dysfunction and cell death [117].

#### 2.4.1. Mitochondrial Unfolded Protein Response

The cell has a quality control pathway to adapt to mitochondrial proteotoxic stress called the mitochondrial unfolded protein response (mtUPR) (Figure 2(f)). mtUPR was first identified as a mitochondrial quality control process over 10 years ago in mammalian cells induced by overexpression of a mutant version of OTC which does not fold properly [118]. This resulted in increased expression of mitochondrial import proteins, folding chaperones and heat shock proteins, and the ATP-dependent mitochondrial protease ClpP [118]. Upregulation of these genes during mtUPR induction was found to occur via activation of the CHOP, MURE1, and MURE2 elements that induces the transcription of a number of proteins including mitochondrial heat shock proteins and other mitochondrial quality control proteins [119], but the mechanisms that lead to the activation of the CHOP, MURE1, and MURE2 remained elusive.

The topic was approached in a simpler model organism, *C. elegans*, by Haynes et al. which facilitated some further characterisation of steps involved in this pathway [120]. mtUPR activation was demonstrated to be dependent on the activity of ATP-dependent mitochondrial protease ClpP [120], suggesting a role for mitochondrial peptides in the process. Further, transcriptional activation of mitochondrial folding chaperones is dependent on nuclear translocation of the ubiquitin-like protein 5 (ubl-5) [121], a bZip transcription factor, and homeodomain containing transcription factor, DVE [122]. Nuclear localisation of this transcription complex is dependent on activity of the mitochondrial peptide transporter, HAF-1, and hence a model was put forth where mitochondrial peptide efflux during proteotoxic stress activates the transcription of mitochondrial heat shock proteins [122].

Although HAF-1 is homologous to mammalian ATP binding cassette proteins, mitochondrially located ATP binding cassette proteins have roles in heme transport, whereas ATP binding cassette proteins with peptide efflux roles are located elsewhere in the cell [123], so thus far mitochondrial peptide export has not been confirmed in mammalian systems. Additionally, upregulation of nuclear encoded mitochondrial protein folding chaperones does not necessarily imply that they actually enter the mitochondrion and get folded themselves. Interestingly, during mtUPR activation, the import efficiency of activating transcription factor associated with stress-1 (ATFS-1) is reduced which allows more to enter the nucleus and activate transcription of mitochondrial quality control proteins [124]. Despite an emerging pathway, the molecular mechanisms by which peptides actually activate assembly of this transcription complex are unclear.

mtUPR is clearly a control process providing cellular adaptation to mitochondrial proteotoxic stress and there is some evidence suggesting a role for the activation of this pathway in chronic disease and ageing. Increased NAD^+^ concentrations have been implicated in longevity, notably through the activation of SIRT proteins or related homologues. Pharmacological increases in NAD^+^, increased hsp-6 reporter activity, and lifespan in *C. elegans* independently of the *C. elegans* SIRT1 homologue [125] reveal mtUPR induction as an additional potential antiageing pathway of NAD^+^ activity. Aged wild type mice display higher Hsp60 protein expression in midbrain than young mice, but this age-dependent effect is diminished in DJ-1 knockout mice [126] providing further evidence that mtUPR induction may be an important adaptation for healthy ageing, as well as implicating mtUPR in PD. Furthermore, overexpression of the mitochondrial chaperone TRAP1 ameliorates *α*-synuclein toxicity [127], which reinforces the idea that upregulation of mitochondrial protein quality control activity may prevent disease, especially PD. Another peculiar piece of evidence that suggests that mtUPR induction is an important adaptation to mitochondrial stress is that the activation of mtUPR appears to increase resistance to the cytotoxic effects of statins, which specifically perturb mitochondrial homeostasis [128]. Interestingly, Hsp60 is overexpressed in cancer cells and prevents cyclophilin D mediated permeability transition and hence promotes cancer cell survival and tumour growth [129]. These findings not only suggest that suppression of mitochondrial apoptosis could be a mechanism by which Hsp60 is pro-survival but also highlight how dysregulation may lead to disease.

#### 2.4.2. Cell-to-Cell Signals

Transcriptomic analysis of ATFS-1-dependent genes demonstrates signaling genes and transcriptional regulators in addition to mitochondrial quality control genes [124], suggesting that mtUPR activation may result in diverse cell signaling pathways. Recent work has also shown that mitochondrial dysfunction in neurons of *C. elegans* induces mtUPR induction in the gut, which provides evidence for cellular adaptations to mitochondrial stress in a noncell autonomous manner [130]. Indeed the implications of this study were that there may be secreted signals that are induced in response to mitochondrial stress and signal to distal tissues. These signaling molecules, termed mitokines, may be proteins involved in retrograde signaling, mitochondrially derived peptides, or perhaps even nucleic acid-based molecules. An example of this phenomena is the peptide humanin, which was protective against AD [131] and believed to be encoded by mtDNA, as it is 99% similar to mitochondrial 16S rRNA and is not present in mtDNA-depleted cells [132]. It has been posited that there may be more mitochondrially derived or induced peptides which could play diverse cellular signalling roles [133]. An example of a mitochondrially induced stress signal comes from a study that showed impaired oxphos function in muscle-caused secretion of fibroblast growth factor 21, hence acting as a mitochondrial stress-responsive hormone [134]. Overall these studies suggest that mitochondrial stress in one tissue may cause adaptive response in other tissues through the secretion of peptides or proteins, and it has also been shown that the mitochondrial genome encodes long noncoding RNA molecules [135]; however, whether these can move out of the mitochondria to function in cell signaling is unknown.

## 3. Conclusions

Mitochondria should not simply be thought of as isolated organelles that generate energy. They have a complex relationship with the rest of the cell requiring back and forth coordination of two genomes and constant communication about the bioenergetic status of the cell. Dysregulation of mitochondrial communication processes and reduced cellular capacity to activate mitochondrial quality control processes in times of stress may therefore be important part of the molecular basis for the mitochondrial component of complex disease.
